# Plasma cortisol-linked gene networks in hepatic and adipose tissues implicate corticosteroid-binding globulin in modulating tissue glucocorticoid action and cardiovascular risk

**DOI:** 10.3389/fendo.2023.1186252

**Published:** 2023-09-06

**Authors:** Sean Bankier, Lingfei Wang, Andrew Crawford, Ruth A. Morgan, Arno Ruusalepp, Ruth Andrew, Johan L. M. Björkegren, Brian R. Walker, Tom Michoel

**Affiliations:** ^1^ University/BHF Centre for Cardiovascular Science, Queen’s Medical Research Institute, University of Edinburgh, Edinburgh, United Kingdom; ^2^ Computational Biology Unit, Department of Informatics, University of Bergen, Bergen, Norway; ^3^ Division of Genetics and Genomics, The Roslin Institute, The University of Edinburgh, Edinburgh, United Kingdom; ^4^ MRC Integrative Epidemiology Unit, University of Bristol, Bristol, United Kingdom; ^5^ SRUC, The Roslin Institute, Edinburgh, United Kingdom; ^6^ Department of Cardiac Surgery, Tartu University Hospital, Tartu, Estonia; ^7^ Department of Cardiology, Institute of Clinical Medicine, Tartu University, Tartu, Estonia; ^8^ Clinical Gene Networks AB, Stockholm, Sweden; ^9^ Department of Medicine, Karolinska Institutet, Karolinska Universitetssjukhuset, Huddinge, Sweden; ^10^ Department of Genetics & Genomic Sciences, Institute of Genomics and Multiscale Biology, Icahn School of Medicine at Mount Sinai, New York, NY, United States; ^11^ Clinical and Translational Research Institute, Newcastle University, Newcastle upon Tyne, United Kingdom

**Keywords:** cortisol, corticosteroid-binding globulin, gene networks, systems genetics, causal inference

## Abstract

Genome-wide association meta-analysis (GWAMA) by the Cortisol Network (CORNET) consortium identified genetic variants spanning the *SERPINA6/SERPINA1* locus on chromosome 14 associated with morning plasma cortisol, cardiovascular disease (CVD), and *SERPINA6* mRNA expression encoding corticosteroid-binding globulin (CBG) in the liver. These and other findings indicate that higher plasma cortisol levels are causally associated with CVD; however, the mechanisms by which variations in CBG lead to CVD are undetermined. Using genomic and transcriptomic data from The Stockholm Tartu Atherosclerosis Reverse Networks Engineering Task (STARNET) study, we identified plasma cortisol-linked single-nucleotide polymorphisms (SNPs) that are trans-associated with genes from seven different vascular and metabolic tissues, finding the highest representation of trans-genes in the liver, subcutaneous fat, and visceral abdominal fat, [false discovery rate (FDR) = 15%]. We identified a subset of cortisol-associated trans-genes that are putatively regulated by the glucocorticoid receptor (GR), the primary transcription factor activated by cortisol. Using causal inference, we identified GR-regulated trans-genes that are responsible for the regulation of tissue-specific gene networks. Cis-expression Quantitative Trait Loci (eQTLs) were used as genetic instruments for identification of pairwise causal relationships from which gene networks could be reconstructed. Gene networks were identified in the liver, subcutaneous fat, and visceral abdominal fat, including a high confidence gene network specific to subcutaneous adipose (FDR = 10%) under the regulation of the interferon regulatory transcription factor, *IRF2*. These data identify a plausible pathway through which variation in the liver CBG production perturbs cortisol-regulated gene networks in peripheral tissues and thereby promote CVD.

## 1 Introduction

The steroid cortisol is the major glucocorticoid hormone involved in mediating the human stress response, with effects on metabolism, cardiovascular homeostasis, and inflammation (1). Excessive cortisol production occurs in Cushing’s syndrome either in response to chronic activation of the hypothalamic-pituitary-adrenal (HPA) axis by increased adrenocorticotropic hormone (ACTH) secretion or through autonomous production of cortisol in an adrenocortical tumor (2). The incidence of Cushing’s syndrome is low, with the number of cases estimated to be between 0.7 and 2.4 cases per million (3). It results in insulin resistance, obesity and hypertension with increased risk of cardiovascular disease (CVD). Similarly, higher plasma cortisol within the population, in the absence of overt Cushing’s syndrome, is associated with risk factors for CVD such as hypertension (4) and type II diabetes (1, 5).

Interindividual variation in plasma cortisol levels has a genetic basis with heritability estimated between 30% and 60% (6). The Cortisol Network (CORNET) consortium conducted a genome-wide association meta-analysis (GWAMA) with the intention of uncovering genetic influences on the HPA axis function (7). This was followed in 2021 with an updated GWAMA of 25,314 individuals across 17 population-based cohorts of European ancestries (8), expanded from 12,597 individuals in the original GWAMA. In an additive genetic model, the new CORNET GWAMA identified 73 genome-wide significant single-nucleotide polymorphisms (SNPs) associated with variation for plasma cortisol at a single locus on chromosome 14. These SNPs were used in a two-sample Mendelian randomization analysis showing that higher cortisol is causative for CVD (8).

The locus on chromosome 14 spans the genes *SERPINA6* and *SERPINA1* that both play roles in the regulation of corticosteroid-binding globulin (CBG), a plasma protein produced in the liver that is responsible for binding 80%–90% of cortisol in the blood (9, 10). *SERPINA6* encodes CBG (11), and *SERPINA1* encodes α1-antitrypsin, an inhibitor of neutrophil elastase, a serine protease that can cleave the reactive center loop of CBG resulting in a 9–10-fold reduction in binding affinity to cortisol (12, 13).

The CORNET GWAMA showed that 21 cortisol-associated SNPs were also cis-expression Quantitative Trait Loci (eQTLs) for *SERPINA6* in the liver and demonstrated that the genetic variation associated with plasma cortisol is driven by *SERPINA6* rather than *SERPINA1* (8). However, although variation in CBG production could explain changes in total plasma cortisol, it is the free fraction of cortisol that is considered to equilibrate with target tissue concentrations and signal through intracellular glucocorticoid receptors (GR) (14, 15). While CBG deficiency may be associated with symptoms (16–18), variations in CBG have not been shown conclusively to influence the tissue response to cortisol in humans.

To test the hypothesis that cortisol-associated genetic variants in the *SERPINA6/SERPINA1* locus influence cortisol delivery to, and hence action in, extrahepatic tissues, we investigated transcriptome-wide associations between cortisol-associated SNPs and gene transcripts across seven different vascular and metabolic tissues from the Stockholm Tartu Atherosclerosis Reverse Networks Engineering Task (STARNET) study (19). As well as conducting a multi-tissue eQTL analysis using STARNET transcriptomics and plasma cortisol-associated SNPs, we identified tissue-specific trans-eQTL-associated genes under the regulation of GR. Moreover, we used a causal inference framework, with cis-eQTLs as genetic instruments, for the reconstruction of causal gene networks within STARNET tissues.

These results provide evidence that genetic variations in CBG production in liver influence extra-hepatic cortisol signaling and provide plausible pathways leading to CVD.

## 2 Materials and methods

### 2.1 Data

STARNET is a cohort-based study of 600 individuals undergoing coronary artery bypass grafting (CABG) for coronary artery disease (CAD) and was used as the primary discovery cohort in this study. These individuals underwent blood genotyping preoperatively for 951,117 genomic markers, and during surgery, seven different tissue samples were obtained and underwent RNA-sequencing (RNA-seq): liver, skeletal muscle, atherosclerotic aortic root, internal mammary artery, visceral abdominal fat, subcutaneous fat, and whole blood. STARNET data are available through a database of Genotypes and Phenotypes (dbGaP) application (accession no. phs001203.v2.p1). A detailed description of data processing can be found in the **Supplemental Material** of this article (section S1.1).

The Stockholm Atherosclerosis Gene Expression (STAGE) study (n = 114) (20) and the Metabolic Syndrome in Man (METSIM) study (n = 982) (21) were used in the replication of causal gene networks identified using STARNET. Gene expression data for the METSIM and STAGE studies are available publicly at Gene expression omnibus (GEO) (accession no. GSE70353 and GSE40231, respectively). Microarray data for the liver, subcutaneous fat, and visceral abdominal fat were used from the STAGE study, and gene expression data from subcutaneous fat were measured in the METSIM study using RNA-seq.

### 2.2 Multi-tissue trans-eQTL discovery

A list of SNPs associated with plasma cortisol was obtained from the summary statistics of the 2021 GWAMA conducted by the CORNET consortium (available at https://datashare.ed.ac.uk/handle/10283/3836) (8). We filtered this list to obtain SNPs that were found to be associated with plasma cortisol at a level of genome-wide significance (p< 5 × 10^-8^) that were taken forward 68 and tested against all genes across STARNET tissues.

The secondary linkage test (P2) is a likelihood ratio test in the Findr package (22) (version 1.0.8) that was used to identify associations between a given SNP (E) and a gene (B) using categorical regression. P2 proposes a null hypothesis where E and B are independent and alternative hypotheses where E is causal for B (E →B). Maximum likelihood estimators are then used to obtain a log likelihood ratio (LLR) between the alternative and null hypotheses. The LLR is then converted to the posterior probability of the alternative hypothesis ${ℋ}_{alt}^{\left(P2\right)}$ being true with empirical estimation of the local false discovery rate (FDR) as a value from 0 to 1 (Equation 1).


(1) $$P\left(E\to B\right)=P({ℋ}_{alt}^{\left(P2\right)}|LLR^{\left(P2\right)}).$$

### 2.3 Identification of glucocorticoid-regulated trans-genes

Multiple datasets were used to identify genes that had prior evidence of putative regulation by GR (23–27). These datasets have been filtered to include targets for NR3C1, the gene that encodes GR.

Trans-genes were categorized according to evidence of GR regulation from datasets shown in **Supplementary Table S1**. Genes were scored against these criteria: 1) appearing in a transcription factor database (ENCODE, TRANSFAC, CHEA); 2) identified as a GR target from chromatin immunoprecipitation sequencing (ChIP-seq) experiment in adipocytes from Yu et al. (23); 3) differentially expressed in response to dexamethasone treatment in adipocytes from Yu et al. (23); and 4) murine homolog of human gene differentially expressed in response to dexamethasone treatment using adrenalectomized mice (FC >1; p-value<0.05) (24). Genes were then ranked according to how well they met the criteria for GR regulation (+1 for each item matched from criteria 1–4).

### 2.4 Causal gene network reconstruction

Pairwise causal inference was used for the reconstruction of cortisol-responsive transcriptional networks across STARNET tissues using cis-eQTL genotypes as genetic instruments with gene expression data from STARNET, as implemented by the Findr software (22). A detailed description of these methods can be found in the **Supplementary Material** of this article (Section S1.2).

### 2.5 Transcription factor target enrichment

Lists of known transcription factor targets for both *NR3C1* and *IRF2* were obtained from ENCODE and TRANSFAC datasets, respectively. These datasets were used to test for an enrichment of known transcription factor targets within novel gene sets derived from gene network targets. This was performed using Fisher’s exact test from the Python module Scipy Stats (28) and involved the creation of a 2 × 2 contingency table based on a tissue-specific background consisting of all genes available in the corresponding tissue.

### 2.6 Gene network replication

Correlations between gene network targets were calculated using gene expression data from STARNET, STAGE, and METSIM. Gene expression matrices were filtered to only include the target genes under investigation. Correlation matrices of corresponding Pearson correlation coefficients as absolute values were constructed in Python.

A background gene set was constructed from the overlapping genes between the STARNET gene expression set that was used for network discovery and the corresponding gene expression set that was being used for replication. The previously described correlation analysis was then repeated using a random set of genes (the same size as the target set) selected from the background gene set. The Kruskal–Wallis test was implemented in Python using Scipy Stats (28) to test if the targeted and randomly sampled correlations follow the same distribution. Both the targeted and random correlations were then plotted as a boxplot using the Python plotting package Seaborn (29).

### 2.7 Gene expression clustering

Hierarchical clustering was performed on correlation values between network targets using the discovery (STARNET) gene expression data and hierarchical clustering from Scipy Stats (28) in Python. The leaves list that resulted from the clustering of the discovery dataset was then extracted and applied to the correlations between target genes from the corresponding replication dataset. Both sets of clustered correlation values were then plotted as opposing correlation heatmaps with Seaborn (29).

## 3 Results

### 3.1 Cortisol-associated trans-genes

SNPs associated with plasma cortisol at the *SERPINA6/SERPINA1* locus have previously been linked as expression single-nucleotide polymorphisms (eSNPs) for *SERPINA6* in the liver (8). Using genotype and tissue-specific RNA-seq data from the STARNET cohort, we explored the hepatic and extrahepatic consequences of genetic variation for plasma cortisol using 73 cortisol-associated SNPs at genome-wide significance (p< 5 × 10^-8^) identified from the CORNET GWAMA (8). We identified 704 eQTL associations in cis and trans between plasma cortisol-associated SNPs and genes measured across all STARNET tissues, composed of 262 unique genes and 72 SNPs at a 15% FDR threshold (**Supplementary Tables S2**, **S3**).

**Figure 1:**
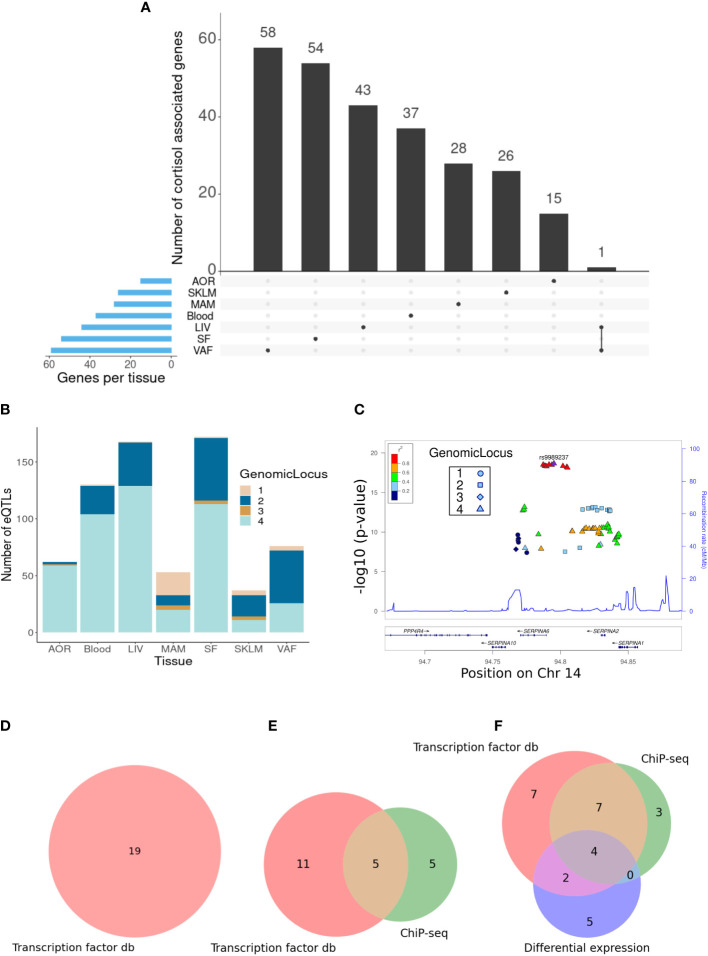
Identification of cortisol-associated trans-genes across STARNET tissues (FDR = 15%). **(A)** Upset plot showing the distribution of trans-genes across STARNET tissues, including genes shared by multiple tissues. Tissues include the atherosclerotic aortic root (AOR), skeletal muscle (SKLM), internal mammary artery (MAM), blood (Blood), liver (LIV), subcutaneous fat (SF), and visceral abdominal fat (VAF). **(B)** Distribution of trans-eQTLs across tissues and colored by genomic locus (LD block) of associated SNP. **(C)** LocusZoom plot (30) showing the location of cortisol-associated SNPs within defined LD blocks. **(D)** Venn diagrams where groupings represent different sources used to identify GR-linked trans-genes in the liver, **(E)** visceral abdominal fat, and **(F)** subcutaneous fat. These sources include transcription factor databases (db), ChIP-seq from perturbation-based experiments (23), and differential expression of dexamethasone-treated mice (24).

The tissues with the greatest number of trans-genes were the liver, subcutaneous fat, and visceral abdominal fat, with a combined total of 157 trans-genes and 422 total SNP–gene associations (FDR = 15%) (**Figure 1A**). The vast majority of trans-eQTL associations were specific to a single tissue. A single trans-gene, the glycosyltransferase-encoding gene *OGT*, was identified in both the liver and visceral abdominal fat. However, as this was the only cross-tissue trans-gene identified, suggesting that the transcriptional impact of genetic variation at the *SERPINA6/SERPINA1* locus is highly tissue-specific. The CORNET GWAMA describes four blocks of SNPs in linkage disequilibrium (LD), which represent the cortisol-associated variation at the *SERPINA6/SERPINA1* locus (8). We observed that LD blocks 2 and 4 represent the majority of the variation across all tissues in the trans-gene sets (**Figures 1B, C**).

### 3.2 GR-regulated trans-genes associated with plasma cortisol

As the GR is the primary mechanism by which cortisol influences transcription, we sought to identify a subset of cortisol-associated trans-genes that were also regulated by the GR. The cortisol-associated trans-genes identified in this study were compared to sets of known GR targets identified from different sources as described in **Supplementary Table S1**. This included large projects such as ENCODE, TRANSFAC, and CHEA that predict transcription factor-binding targets from high-throughput transcription factor-binding assays. We also included predicted GR targets from perturbation-based experiments in specific tissues. ChIP-seq and microarray analysis has been used to identify 274 glucocorticoid-regulated genes in 3TS-L1 adipocytes, a murine-derived cell line (23). In addition, RNA-seq data in subcutaneous fat from adrenalectomized mice treated with dexamethasone, a GR agonist, have been used to identify genes that are differentially expressed (24).

The greatest number of unique cortisol-associated trans-genes was identified in the liver (n = 43), subcutaneous fat (n = 54), and visceral abdominal fat (n = 59) at a 15% FDR threshold. The involvement of these tissues in glucocorticoid signaling and physiological effects has been well documented in the literature (31–34); therefore, the identification of GR-regulated trans-genes was restricted to these tissues. Comparisons of genes identified as glucocorticoid-regulated in 3T3-L1 adipocytes were only made with subcutaneous and visceral adipose trans-genes. Likewise, as the murine RNA-seq experiments were restricted to subcutaneous adipose, only subcutaneous adipose trans-genes were compared to these differentially expressed genes.

In the liver trans-gene set, 19/43 genes were identified that were present in either the ENCODE, TRANSFAC, or CHEA datasets (FDR = 15%) (**Figure 1D**; **Supplementary Table S4**). This includes *SERPINA6* that is cis-associated with genetic variation for plasma cortisol, as described previously (8). One gene, *CPEB2*, was identified in more than one dataset and was present in both ENCODE and CHEA. *CPEB2* (posterior probability = 0.89) is a regulator of translation, splice variants of which have been linked to cancer metastasis (35).

Visceral adipose tissue had the largest number of cortisol-associated trans-genes. Here, 21/59 of these genes had some evidence of being targets of GR (**Figure 1E**; **Supplementary Table S5**). There were five genes that had been identified as GR targets from both high-throughput transcription factor-binding assays and adipose-specific experiments. These include *CD163* and *LUC7L3*. *CD163* is a hemoglobin scavenger protein that is expressed in macrophages and involved in the clearance of hemoglobin/haptoglobin complexes that may play a role in the protection from oxidative damage. It also plays a role in activating macrophages as part of the inflammatory response (36). *LUC7L3*, also known as CROP, encodes a protein that is involved in alternative splicing and is associated with human heart failure (37). It has also been shown to play a role in the inhibition of hepatitis B replication (38).

Of the cortisol-associated trans-genes identified in subcutaneous adipose (FDR = 15%), 28/54 genes were either present in a transcription factor dataset or identified from the adipose-specific perturbation datasets (**Figure 1F**; **Supplementary Table S6**). There were 13 genes that had been identified as GR targets from both high-throughput transcription factor-binding assays and adipose-specific experiments. These include *RNF13* that encodes IRE1α-interacting protein that plays an important role in the endoplasmic reticulum (ER) stress response through regulation of IRE1α, a critical sensor of unfolded proteins (39). Also *IRF2*, encoding the transcription factor Interferon Regulatory Factor 2 that plays an important role as a repressor of *IRF1* that in turn is involved in the interferon-mediated immune response (40). Furthermore, *IRF1* has previously been identified as a marker for glucocorticoid sensitivity in peripheral blood (41).

### 3.3 Reconstruction of cortisol-associated gene networks

Having identified cortisol-associated trans-genes that are regulated by GR, causal estimates were obtained for pairwise relationships between GR-regulated trans-genes and all other genes within the given tissue. This was carried out for all GR-regulated trans-genes in the liver, subcutaneous fat, and visceral abdominal fat with a valid cis-eQTL instrument (12, 19, and 7 genes, respectively) (**Supplementary Table S7**). A 10% global FDR threshold was then imposed for each gene set (**Table 1**). Primary networks were obtained by filtering to include only GR trans-genes with a minimum of four target genes at the global FDR threshold.

**Table 1:** Number of network targets following FDR filtering.

Tissue	FDR threshold	Total targets	Network regulator	Regulator targets
Liver	15%	197	*CPEB2*	190
	10%	48	*CPEB2*	44
Subcutaneous fat	15%	1,701	*RNF13*	416
			*IRF2*	247
			*PBX2*	883
	10%	486	*RNF13*	215
			*IRF2*	128
			*PBX2*	138
Visceral abdominal fat	15%	396	*CD163*	378
			*LUC7L3*	15
	10%	17	*CD163*	4
			*LUC7L3*	11

Total targets include all pairwise interactions at the given threshold, and network regulators correspond to trans-genes with at least four network targets at the given FDR threshold. Inclusive of network regulators present at both 10% and 15% thresholds.

**Figure 2:**
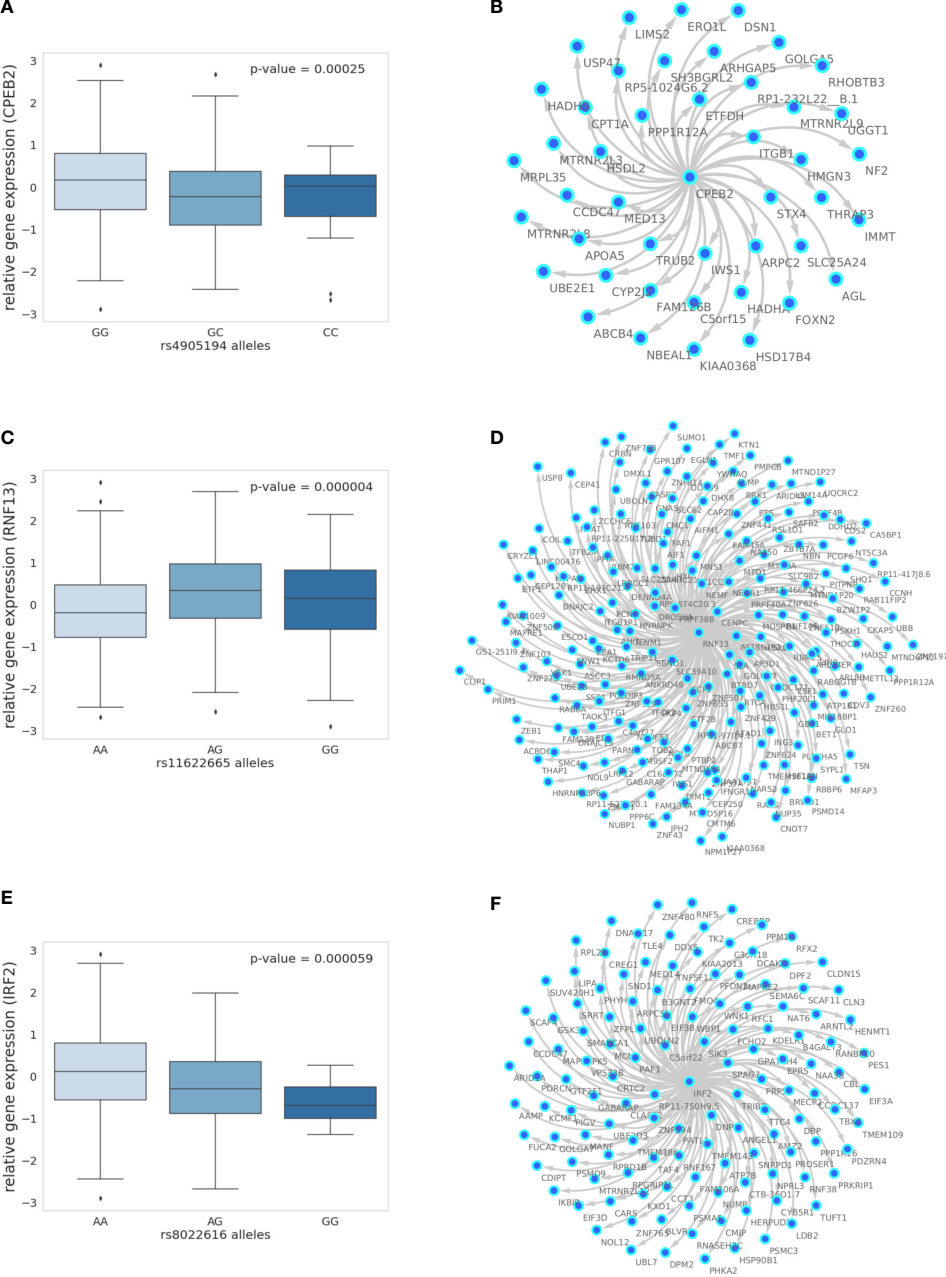
The 10% FDR gene networks in STARNET across different tissues. **(A)** Gene expression boxplot in the liver showing trans-association with cortisol-linked SNP rs4905194 and *CPEB2*, **(B)** in subcutaneous fat between rs11622665 and *RNF13* and **(C)** rs8022616 and *IRF2* (p-value obtained from Kruskal–Wallis test statistic). Box shows quarterlies of the dataset, with whiskers indicating the upper and lower variability of the distribution. **(D)** Causal gene network reconstructed from pairwise interactions from GR-regulated trans-genes against all other genes in the corresponding tissue for *CPEB2*, **(E)** *RNF13*, and **(F)** *IRF2*. Edges represent Bayesian posterior probabilities of pairwise interaction between genes (nodes) exceeding 10% global FDR. Arrow indicates direction of regulation, and interactions were only retained where parent node had at least four targets.

In the liver, we identified a single gene network driven by *CPEB2*, which was found to be trans-associated with the cortisol-associated SNP rs4905194 (**Figure 2A**). This network contained 48 causal interactions driven by *CPEB2* at a 10% FDR threshold (**Figure 2D**; **Supplementary Table S9**). It is notable that *CPEB2* appears as the only network regulator in the liver considering it was also the cortisol-associated trans-gene with the strongest links to GR regulation from the liver trans-gene set. A detailed description of the *CPEB2* network and all other networks identified can be found in the **Supplementary Information** (Section S2.1).

In subcutaneous fat, two major subnetworks were identified under the regulation of the genes *RNF13* and *IRF2*. This includes a total of 343 causal relationships across both subnetworks, including two genes shared by both subnetworks. *RNF13* was found to be trans-associated with the cortisol-associated SNP rs11622665 (**Figure 2B**) and represents the largest subcutaneous fat subnetwork with 215 gene targets at a 10% FDR threshold (**Figure 2E**; **Supplementary Table S10**).

The transcription factor *IRF2*, which was associated with the cortisol-linked SNP rs8022616 (**Figure 2C**), was found to putatively regulate a network of 128 genes (FDR = 10%) (**Figure 2F**). Some notable targets of *IRF2* include *LDB2* (posterior probability = 0.94) and *LIPA* (posterior probability = 0.91). GWAS suggests functions for *LIPA* related to CAD and ischemic cardiomyopathy (42), while *LDB2* has been demonstrated to be involved in the development of atherosclerosis (43). Additionally, cortisol has been shown to induce a 5-fold reduction in *LDB2* expression in adipocytes (44).

Predicted *IRF2* transcription factor targets have been previously described as part of the TRANSFAC dataset. We examined the overlap between predicted *IRF2* targets in TRANSFAC, and gene targets within the *IRF2* causal networks were identified in subcutaneous fat. A true network of *IRF2* targets would be expected to show an enrichment of predicted *IRF2*. Using Fisher’s exact test on data from subcutaneous fat, at a 10% FDR threshold, the *IRF2* network had 128 target genes, 35 of which were also predicted *IRF2* targets (p = 0.08); at a 15% FDR threshold, 104/247 causal targets were also predicted targets of *IRF2* in TRANSFAC (p = 0.005). Decreasing the global FDR beyond this threshold increased the number of TRANSFAC targets within the pool of causal targets, however at a lower enrichment (p = 0.046) (**Supplementary Table S12**).

In addition to examining the prevalence of *IRF2* targets within the *IRF2* causal network, we investigated the overlap between network genes that are also regulated by GR. We observed an enrichment of ENCODE GR targets at 15% and 20% FDR thresholds (p< 0.05) including 68 and 138 GR targets, respectively. No GR enrichment was observed in either CHEA or TRANSFAC datasets for *IRF2* networks.

### 3.4 Co-expression of cortisol network targets in independent datasets

Causal gene networks represent coordinated changes in gene expression in response to changes in the expression of network regulators. Therefore, it is possible to examine if these changes in gene expression are present in independent datasets using gene expression data alone. We used RNA-seq and microarray data from the METSIM and STAGE datasets, respectively, to compare patterns in gene expression within causal networks predicted from STARNET. As METSIM only contains gene expression data for subcutaneous fat, analysis was restricted to the causal networks identified in STARNET subcutaneous fat.

Absolute correlation coefficients between the targets of the previously described network regulators were calculated, and their distributions were compared to distributions of random sets of genes selected from the replication gene expression data, the same size as the corresponding target gene set. The difference between targeted and random distributions was formalized using the Kruskal–Wallis test for each subnetwork (**Table 2**).

**Table 2:** Correlations between network targets within replication datasets.

Replication dataset	Tissue	Network regulator	p-value	No. target genes
METSIM	Subcutaneous fat	*IRF2*	< 1.0×10^-300^	128
		*RNF13*	2.3×10^-7^	215
STAGE	Liver	*CPEB2*	8.2×10^-32^	44
	Subcutaneous fat	*IRF2*	8.3×10^-86^	128
		*RNF13*	< 1.0×10^-300^	215
	Visceral abdominal fat	*CD163*	2.6×10^-3^	4
		*LUC7L3*	4.4×10^-1^	11

The Kruskal–Wallis test calculated for the distribution of correlations between network targets compared to correlations within random gene sets of the same size.

**Figure 3:**
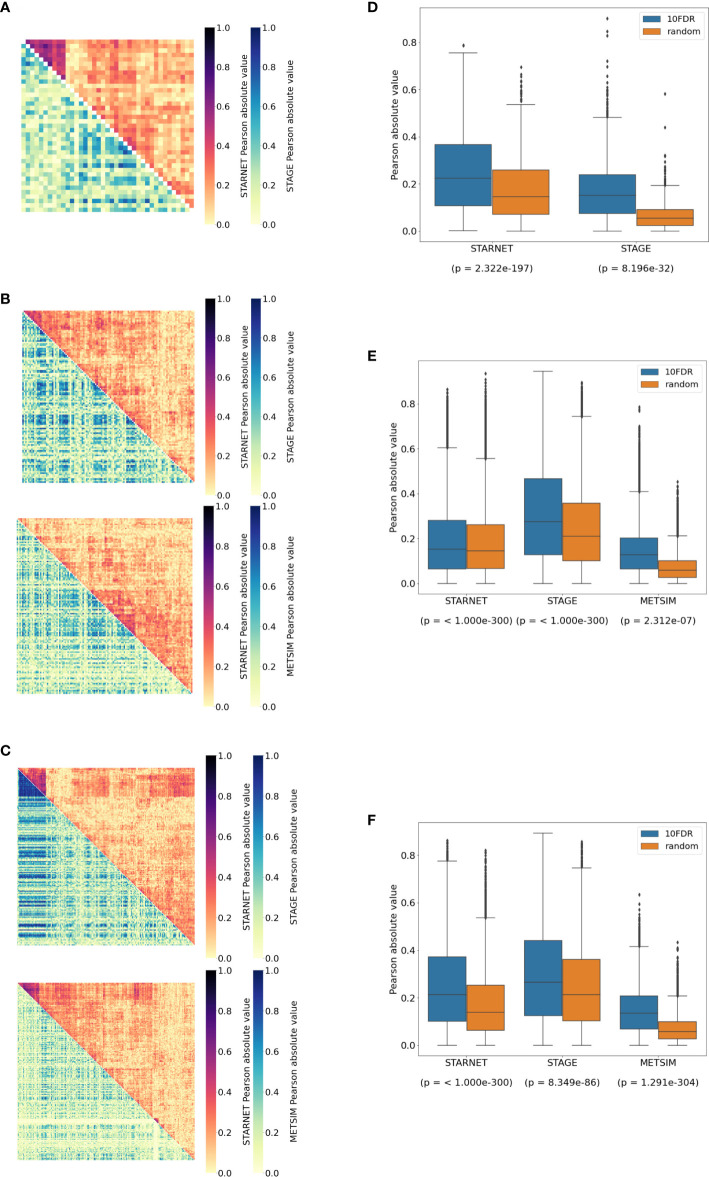
Replication of cortisol-associated gene networks in independent datasets. **(A)** Correlation heatmap showing pairwise Pearson correlations between *CPEB2*, **(B)** *IRF2*, and **(C)** *RNF13* network targets. Hierarchical clustering of genes in STARNET (discovery) was applied to the same genes within replication datasets. **(D)** Correlations between network targets in discovery vs. replication datasets for *CPEB2*, **(E)** *IRF2*, and **(F)** *RNF13* networks. The Kruskal–Wallis test calculated for the distribution of correlations between network targets compared to correlations within random gene sets of the same size.

In the liver, correlations between network targets of the single subnetwork under the regulation of *CPEB2* were observed in STARNET and STAGE. Hierarchical clustering within the STARNET liver also revealed clustering of correlated genes that were retained when the clustered gene order was then applied to the STAGE liver (**Figure 3A**). Correlations between the 44 *CPEB2* target genes in the STAGE liver were stronger than their random counterparts (p = 8.2 × 10^-32^), with this shift also being observed in the STARNET liver (p = 2.32 × 10^-197^) (**Figure 3D**).

In subcutaneous fat, correlations were observed between the network targets of *RNF13* and *IRF2*, and hierarchical clustering patterns from STARNET were applied to the replication datasets of STAGE and METSIM (**Figures 3B, C**). For *RNF13*, similar patterns of co-expression were observed in the STAGE subcutaneous fat following clustering; however, this was not the case in the METSIM dataset (**Figure 3B**). Despite this, *RNF13* targets appeared more highly correlated than their randomly selected counterparts in STARNET (p< 1.0 × 10^-300^), STAGE (p< 1.0 × 10^-300^) and to a lesser extent in METSIM (p = 2.3 × 10^-7^) (**Figure 3E**).

In subcutaneous fat, patterns of co-expression between *IRF2* targets were conserved most prominently in METSIM; however, co-expression was less strongly correlated compared with *RNF13* targets (**Figure 3C**). *IRF2* subcutaneous fat subnetwork targets were more strongly correlated than their random counterparts in STARNET (p< 1.0 × 10^-300^), STAGE (p = 8.35 × 10^-86^), and METSIM (p< 1.0 × 10^-300^) (**Figure 3F**).

## 4 Discussion

In this study, we have characterized the impact that genetic variation for plasma cortisol has upon tissue-specific gene expression. We showed that cortisol-linked genetic variants at the *SERPINA6/SERPINA1* locus mediate changes in gene expression in trans across multiple tissues, in addition to the cis-associations in the liver that have been described previously (8). We have scrutinized these trans-associations to identify a subset of genes that are regulated by glucocorticoids and in turn regulate downstream transcriptional networks, thus providing a deeper understanding of the transcriptional landscape driven by cortisol-linked genetic variation that may underpin the progression to CVD.

CBG, as encoded by *SERPINA6*, is responsible for binding cortisol in the blood. It has remained uncertain whether variation in CBG impacts the availability of cortisol within tissues, since any resulting change in free cortisol concentrations would be expected to be adjusted by negative feedback of the HPA axis (45). However, deleterious mutations in CBG are associated with dysfunction in animals and humans, suggesting an impact of CBG on cortisol signaling (45). Our major finding that downstream transcriptomic changes in extrahepatic tissues are associated with genetic variation at the *SERPINA6* locus lends strong support to the hypothesis that CBG influences tissue delivery of cortisol and modulates glucocorticoid-induced changes in gene expression.

For the STARNET study, whole-blood samples were taken preoperatively and all other tissues including the liver were taken during the CABG surgery. In addition to any rise in cortisol due to anxiety and disturbed sleep in anticipation of surgery, the human stress response to surgery has been well characterized and results in stimulation of the HPA axis leading to high levels of cortisol in the blood both during and post-surgery (46). Surgery is also associated with a very rapid fall in CBG production. Therefore, it is uncertain if cortisol-associated gene expression patterns observed in STARNET would also be observed in an unstressed healthy population. It may be that CBG influences the dynamic range of alterations in free plasma cortisol during stress rather than affecting the delivery of cortisol to tissues in unstressed conditions. However, considering that co-expression of the network targets was reproducible within independent samples from the METSIM study, obtained under nonsurgical conditions, this suggests that the cortisol-associated networks we inferred from STARNET do operate also in unstressed conditions.

The tissues with the greatest number of trans-genes identified were the liver and both subcutaneous and visceral abdominal fat, all tissues known to play a role in glucocorticoid biology. In the liver, glucocorticoids have extensive effects on glucose and fatty acid metabolism (31, 32), while in adipose tissue, glucocorticoids regulate lipogenesis and lipid turnover (33, 34). Skeletal muscle is also a major target of glucocorticoids, where they modulate protein and glucose metabolism (47). A lack of available data for identifying tissue-specific GR targets in other tissues means that potential GR targets may have been missed in tissues outside of the liver and adipose.

We identified a subset of GR-responsive genes in the liver, subcutaneous fat, and visceral adipose fat. However, we did not observe a statistical enrichment of GR-regulated genes in any of these trans-gene sets. This does not negate the identification of GR targets that are associated with plasma cortisol, but it may imply that there are some effects of cortisol-linked genetic variation that are mediated by mechanisms other than directly by GR either through secondary regulation by GR-regulated genes or through the alternative mineralocorticoid receptor. Indeed, some of the genes with higher levels of evidence for GR regulation also demonstrated regulation of transcription networks, e.g., *CPEB2*, *IRF2*, and *RNF13*. This supports our strategy of setting a relatively lenient FDR threshold and then filtering to identify cortisol-associated trans-genes with prior evidence of GR regulation.

It should be noted that different FDR thresholds were used for the trans-gene discovery and for the network reconstruction. Initially, we selected a more lenient threshold of 15% for the identification of trans-genes, considering that trans-eQTLs tend to exhibit weaker associations compared to their cis counterparts (47). We then decided to restrict our list of trans-associations by implementing a biological rather than a statistical threshold, limiting the number of trans-genes to those with evidence of GR regulation. However, given that there was no biological threshold implemented with network reconstruction, a more stringent FDR threshold was appropriate. The 10% FDR in this context implies that 1 in 10 edges of a given network is a potential false positive. However, given the strength of the replication within independent datasets, this suggests that these networks are considerably robust.

We identified causal gene networks in the liver, subcutaneous fat, and visceral abdominal fat where cortisol-associated trans-genes act as regulators of subnetworks within overarching tissue-specific networks. Pairwise causal relationships were established between network regulators and downstream targets using cis-eQTLs as genetic instruments. This approach has the benefit of generating directed relationships between a regulator and target while accounting for any unobserved confounding. However, a drawback of this approach is that we are limited by only being able to examine GR-regulated trans-genes with valid cis-eQTLs. This means that there could be valid cortisol-responsive networks regulated by GR trans-genes that we were unable to predict due to lack of a corresponding instrument.

*IRF2* stands out as a network regulator of particular interest. There is strong evidence of GR regulation, where *IRF2* has been identified as a GR target from published dexamethasone-treated adipocyte ChIP-seq experiments (23) and as a putative GR target within ENCODE. It is robustly associated with its corresponding cis-eQTL instrument, and there is an enrichment of *IRF2* targets within our predicted *IRF2*-regulated causal network. Additionally, we show evidence of regulation by glucocorticoids within the targets of *IRF2*, potentially suggesting evidence of a feed-forward loop motif (48). Interestingly, the genotype for rs8022616, the cortisol-associated SNP linked to *IRF2* expression in subcutaneous fat, is associated with a decrease in *IRF2* expression. Previous evidence suggests that interferon signaling is inhibited by glucocorticoids (49, 50).

Although we have determined the direction of causality between the regulator and target genes, we do not know if the expression of the target gene is upregulated or downregulated in response to modulation of the regulator. This could be investigated through functional experiments within a relevant cell line, whereby the differential gene expression of target genes is measured in response to perturbation of the network regulator. To take this one step further, the results of a cell line experiment could be used to determine the dynamics of the putative cortisol networks using systems biology approaches for modelling gene expression (51).

In conclusion, we have linked genetic variation for plasma cortisol to changes in gene expression across the genome, beyond that which has been previously described at the *SERPINA6/SERPINA1* locus (8) and extending to adipose tissue as well as the liver. Furthermore, we have shown that a subset of these trans-genes is driven by the GR and in turn drives transcriptional networks across different tissues. These networks have been found to be robust and their network targets appear co-expressed within independent gene expression datasets of the same tissue. Further study of these networks and their downstream targets could be used to enhance our mechanistic understanding of the pathways linking cortisol with complex diseases as described in observational studies.

## Data availability statement

All code used in the analyses presented in this study are available at the following repository: https://github.com/sbankier/cortisol_networks/tree/main. Data from the Stockholm Tartu Atherosclerosis Reverse Networks Engineering Task study (STARNET) are available through a database of Genotypes and Phenotypes (dbGaP) application (accession no. phs001203.v2.p1). Gene expression data from The Stockholm Atherosclerosis Gene Expression study (STAGE) and the Metabolic Syndrome in Man study (METSIM) are available publicly at GEO (accession no. GSE70353 and GSE40231, respectively). The summary statistics from the CORNET GWAMA are available at Edinburgh DataShare: https://datashare.ed.ac.uk/handle/10283/3836.

## Ethics statement

The studies involving human participants were reviewed and approved by ethical approvals: Tartu, Dnr 154/7 and 188/M-12, Mount Sinai, IRB-20-03781. The patients/participants provided their written informed consent to participate in this study.

## Author contributions

SB, TM, and BW contributed to the conception and design of this research. SB conducted all formal analyses and visualizations and wrote the article, supervised by TM, BW, and RA. LW and TM developed and supported the use of and interpretation of outputs from the software Findr. AC contributed to data analysis and interpretation for the CORNET consortium. RM conducted the experiments and contributed to data analysis of dexamethasone-treated mice. AR and JB provided access to and contributed to interpretation of data from the STARNET cohort. All authors reviewed the article and approved the submitted version.
