# Supplementary material for: Plasma cortisol-linked gene networks in hepatic and adipose tissues implicate corticosteroid-binding globulin in modulating tissue glucocorticoid action and cardiovascular risk
Source: Front Endocrinol (Lausanne). 2023 Sep 6;14:1186252. doi: 10.3389/fendo.2023.1186252 (PMC10513085; doi:10.3389/fendo.2023.1186252)
Supplement: Supplementary file 1 [file DataSheet_1.pdf]

Supplementary material

S1 Supplementary methods

S1.1 Processing of genotype and tissue-specific gene expression data

All STARNET genotype and gene expression data obtained for this project had undergone both Quality Control (QC) and normalisation as described previously[1]. The Human OmniExpressExome-8v1 bead chip was used with GRCh37 and contains 951,117 genomic markers and imputed to 12,450,918 autosomal SNPs. Genotypes were contained in matrices within the -012 format and a filtering step was included to remove any SNPs which had missing values for any samples and to exclude any SNPs which had a Minor Allele Frequency (MAF) < 5% (6245505 SNPs).

Gene expression for STARNET tissue samples was measured using RNA-seq. RNA samples with less than 1M uniquely mapped reads were excluded, which removed 12 samples with extremely low read counts. The read counts of the samples used in the final analysis were between 15-30 million reads (Figure S1).

The numbers of samples and genes retained can be seen in Table S13. Having obtained gene expression matrices from Franzen *et al*, we conducted Principal Component Analysis (PCA) to confirm that there were no outliers within the samples (Figure S2). Ensembl Biomart (GRCh37) was used to label transcripts (provided as Ensembl IDs) with gene name, chromosome location, gene start, gene end and the Transcription Start Site (TSS).

METSIM gene expression data were obtained as Transcripts Per Million (TPM) and showed an inflation of higher correlated genes from the normal distribution (Figure S3). To account for this, the METSIM gene expression values were log2 transformed (TPM+1) followed by a re-running of the PCA. The log2 transformed expression values were then fitted to a linear model in R, while adjusting on the 1st principal component. The residuals of this model replaced the count values that were used in all subsequent analyses and no longer showed inflated correlation values (Figure S3).

S1.2 Causal gene network reconstruction

Cis-eQTL discovery was carried out to identify genetic instruments to be used for causal inference analysis with Findr[2] (Figure S4). An automated pipeline was established to use the secondary linkage test (P2) to calculate SNP-gene associations when supplied with a list of genes. SNP-gene associations were obtained between all SNPs within 1 Mb of the trans-gene and all other transcripts using the same tissue dataset as the trans-gene.

Associations between all SNPs and the trans-gene were extracted from the output. A primary cis-eQTL was selected for each gene, defined as the SNP-gene association with the highest Findr P2 score for the trans-gene. An alternate, independent, cis-eQTL was selected as the second strongest cis-association not in LD with the primary cis-eQTL. LD between SNPs was calculated as the Pearson correlation coefficient between the primary cis-eQTL genotype and all other SNP genotypes. The alternate cis-eQTL was defined as the top cis-association, which was not in LD with the primary cis-eQTL ($R^2 < 0.5$).

To test for pleiotropy between the selected instrument and other cis-genes, cis associations between all cis genes (± 1 Mb of the trans-gene) and the primary instrument were obtained, as detailed in supplementary results (section S2.2)

All genes with a valid cis-eQTL (P2 > 0.75) were taken forward for causal analysis with Findr. Causal relationships were inferred between these cis-eQTL genes (A-genes) and all other transcripts expressed in the same tissue (B-genes). The input was as follows: (dg) array of eQTL genotypes A-gene in 012 format, (d) array of normalised A-gene expression levels, (dt) array of expression levels for all B-genes in the relevant tissue sorted with d appearing on top.

The output of all tests in Findr was calculated using the *pijs_gassist* function from the Findr Python package. The posterior probability of a causal interaction ($P(A \rightarrow B)$) was calculated from the product of the alternative hypotheses from the secondary linkage test (P2) and the controlled test (P5). The controlled test (P5) is a likelihood ratio test, which can be used as a composite test with secondary linkage (P2*P5) to infer a causal $A \rightarrow B$ relationship while using a cis-eQTL, E, as an instrumental variable. P5 examines whether A and B are not associated independently with E (i.e. whether they are still coexpressed after adjusting for E), while P2 tests for a direct association between $E \rightarrow B$. Previous work has demonstrated that most cis-eQTLs are only associated with a single gene[3], therefore selecting cis-eQTLs specifically as an instrument allows $E \rightarrow B$ to be used as a proxy for estimating causal effects between $A \rightarrow B$. When combined with P2, P5 can then be used to account for the comparatively few instances where E is a cis-eQTL for more than one

gene, although in such cases a false positive may still occur when A and B are confounded by a common regulator[2,4]. Therefore, we also examined manually all cis-associations for selected E of interest, to account for any sources of pleiotropy that may have been missed by P5 (section S2.2).

This approach was undertaken for each A-gene in a given tissue in a iterative fashion. Following completion of analysis for all A-genes in a tissue, the output was converted from the default matrix format to a Pandas DataFrame. Each tissue-specific gene set of A $\rightarrow$B pairwise interactions was filtered according to a local precision FDR threshold (Findr score) for each interaction, to correspond to a global FDR for all interactions in the tissue set.

Networks were assembled, using the network visualisation tool, Cytoscape (version 3.8.0), from FDR thresholded pairwise gene interactions previously described. These were assembled as directed networks where the A-gene acts as the parent node and the B-gene as the child node, with the posterior probability of an A $\rightarrow$B interaction forming the network edge.

The Findr score for a given A $\rightarrow$B pairwise interaction, or E $\rightarrow$B in the case of P2 testing (Table S3), is calculated as 1 minus the probability of that interaction being a false positive. To obtain the probability of a false positive across all interactions in a gene set, this was calculated as 1 minus the mean of all local precision FDR scores for a given tissue. A Findr score cut off was then set to obtain interaction sets at 10%, 15% and 20% global FDR thresholds[5].

S1.3 Functional annotation and clustering for GO enrichment

Gene sets were functionally annotated using the Database for Annotation, Visualization and Integrated Discovery (DAVID)[6]. This web-based application allows for the generation of gene clusters that have been grouped in relation to an enrichment of functional terms, including but not limited to Gene Ontology (GO) terms. The strength of the gene-term interactions are measured by EASE scores, a modified Fisher's exact test. An enrichment score for a given cluster is generated as the geometric mean of all the EASE scores within a cluster that has undergone -log transformation. For all analyses Ensembl Gene IDs were used as the input format for DAVID as opposed to universal gene symbols.

For the analyses conducted, all of the default annotation options were selected in addition to: GAD DISEASE, GO TERM BP FAT, GO TERM CC FAT, GO TERM MF FAT, PUBMED ID, REACTOME PATHWAY, BIOGRID INTERACTIONS and UP TISSUE. Gene sets were then run using DAVID

and functionally enriched clusters generated using high classification stringency. Tissue-specific gene sets from STARNET RNA-seq datasets were used as background for enrichment (Table S13).

S2 Supplementary results

S2.1 Detailed networks description

S2.1.1 Liver

A trans-association was identified between cortisol-associated SNP rs4905194 and *CPEB2* in STARNET-liver (Figure 2A). Following cis-eQTL discovery for *CPEB2*, a SNP peak was identified upstream of the *CPEB2* TSS represented by the instrument rs62410848, which was used as an instrumental variable for network reconstruction (Figure S5).

48 causal interactions were obtained at a global 10% FDR threshold (Posterior probability > 0.855) (Table S9). When filtering to a minimum of 4 targets, the only GR-regulated trans-gene that remained was *CPEB2* (Figure 2E). Notably, this was also the trans-gene that appeared in the most GR target datasets, forming a network with 44 target genes. Functional enrichment was performed using DAVID for all *CPEB2* target genes (Table S8). The strongest cluster was related to fatty acid beta oxidation and lipid metabolism, including 5 genes related to GO:0006635 - fatty acid beta-oxidation (adj p-value = 0.002). Other enrichments stem from 8 genes related to acquired immunodeficiency syndrome and disease progression (adj p-value = 0.003).

The strongest causal relationship within this network was between *CPEB2* and the gene *HADHA* (Posterior Probability = 0.99), responsible for encoding the alpha subunit of the mitochondrial trifunctional protein[7]. Mutations affecting this protein have been linked to long-chain 3-hydroxyacyl-CoA dehydrogenase (LCHAD) deficiency, which affects the ability to metabolise fatty acids in the liver[8]. These mutations have also been linked to maternal acute fatty liver during pregnancy[9].

S2.1.2 Subcutaneous fat

In STARNET subcutaneous fat, 486 causal relationships were detected at a 10% FDR threshold (Posterior probability = 0.87), which is the most out of all tissues examined (Table S10). When filtering to exclude trans-genes with less than 4 targets at this threshold, 2 major sub-networks are

represented under the regulation of the genes *RNF13* and *IRF2*. This includes a total of 343 causal relationships across both sub-networks, including two genes shared by both sub-networks.

RNF13 was found to be trans-associated with the cortisol-linked SNP rs11622665 (Figure 2E). A cis-eQTL peak of SNPs associated with *RNF13* in STARNET subcutaneous fat was identified upstream of the *RNF13* TSS, represented by the lead SNP rs9853321 which was used as a causal instrument in the reconstruction of the causal network driven by *RNF13* (Figure S5).

RNF13 represents the largest subcutaneous fat sub-network with 215 gene targets at a 10% FDR threshold (Figure 2F). The strongest functional enrichment term for *RNF13* targets is related to Poly(A) RNA binding, where 33 targets are included for this term, GO:0044822 poly(A) RNA binding (adj p-value = 0.01), and 39 targets are included for RNA binding, GO:0003723 RNA binding (adj p-value = 0.04). Other notable terms include 23 genes related to Zinc finger motifs (adj p-value = 0.05).

IRF2 was found to be associated with the cortisol-linked SNP rs8022616 (Figure 2C). Cis-eQTL discovery revealed associations between rs34985265 and *IRF2* expression in subcutaneous fat to obtain an instrument that could be used for causal network reconstruction (Figure S5).

The *IRF2* sub-network contains 128 targets at a 10% FDR threshold (Figure 2D). Following functional enrichment of *IRF2* targets, the strongest enrichment term included 19 genes related to Poly(A) RNA binding (p-value = 0.009), however this association was not retained following multiple testing correction. Some notable targets of *IRF2* include *LDB2* (Posterior probability = 0.94) and *LIPA* (Posterior probability = 0.91). GWAS suggests functions for *LIPA* related to CAD and ischaemic cardiomyopathy and *LDB2* has been demonstrated to be involved in the development of atherosclerosis[10]. Additionally, cortisol has been shown to induce a 5-fold reduction in *LDB2* expression in adipocytes[11].

An additional subcutaneous fat gene network was identified for the transcription factor *PBX2* containing 138 targets at a 10% FDR threshold. However, the cis-eQTL instrument that was used to reconstruct this network was found to be associated with many other genes at the *PBX2* locus, which include causal targets within the *PBX2* network. This indicates that *PBX2* is not independently linked to this instrument and the *PBX2* causal network could be driven by a cis-gene other than *PBX2*.

S2.1.3 Visceral abdominal fat

In STARNET visceral abdominal fat, trans-associations were identified for the genes *CD163* and *LUC7L3* with the same cortisol associated SNP rs2005945 (Figure S6A-B). Although STARNET-visceral abdominal fat contained the largest number of trans-associations with cortisol SNPs, the fewest causal relationships were detected in this tissue at 10% FDR (Table S11). Two small sub-networks were detected, regulated by the genes *LUC7L3* and *CD163* composed of eleven and four targets (Figure S6C). Interestingly, when the FDR threshold is reduced to 15% the sub-network for *CD163* is expanded to include 378 targets, a much more dramatic expansion compared to reducing the threshold to 15% FDR with other regulators. The networks for *CD163* and *LUC7L3* were identified using the cis-eQTLs rs73059776 and rs6504682, respectively (Figure S6D). Due to the small size of the 10% FDR networks, functional enrichment and clustering was not carried out for either of the networks identified in visceral abdominal fat.

S2.2 Application of independent genetic instruments for gene network reconstruction

To study the impact of instrument selection on the reconstruction of causal networks we examined the distribution of local cis-eQTLs for each of the GR-regulated trans-genes that was found to regulate a network. Primary instruments were selected as the strongest cis-eQTL within a 1 Mb window of the associated gene, as determined by secondary linkage test posterior probability. However, the landscape of gene expression-linked genetic variation can involve several loci associated with the expression of the same gene to differing degrees. In addition to selecting a primary cis-eQTL as an instrument, alternate independent instruments were also identified. These were defined as the second strongest cis SNP-gene association which was not in LD with the primary instrument ($R^2 < 0.5$) (Figure S5).

Causal relationships in STARNET liver were defined by a GR-regulated network under the regulation of *CPEB2* (FDR = 10%). The genetic instrument used to construct this network, rs62410848 (posterior probability = 0.90), is the strongest cis-eQTL for *CPEB2*, located less than 100 Kb upstream of the *CPEB2* locus. An independent peak was identified 400 Kb upstream of *CPEB2*, represented by rs6847363 as the top cis-association in this region (posterior probability = 0.48). As this independent instrument fell below the required threshold (posterior probability > 0.75), causal analysis was not carried out using rs6847363 as an instrument.

To determine the robustness of the primary instrument, we examined cis-associations with other genes within this locus (± 1 Mb). While *CPEB2* was the strongest cis-eQTL association in this region, rs62410848 was also seen to be associated with *CD38* (posterior probability = 0.85), a gene ~800 Kb downstream of *CPEB2*. Although *CD38* is not associated with any cortisol variants at the *SERPINA6*/*SERPINA1* locus, it has been identified as being regulated by glucocorticoids in smooth muscle cells[12] and has been identified as a GR target in ENCODE. However, *CD38* does not appear as a target of *CPEB2*, which suggests a low P5 score. This suggests that *CPEB2* and *CD38* are independently associated with rs62410848 and that *CPEB2* is the true network regulator in this cis region.

In STARNET subcutaneous fat, the *IRF2* sub-network was generated using the SNP rs34985265 (posterior probability = 0.94) located ~500 Kb upstream of *IRF2*. The strongest independent cis-eQTL for *IRF2*, rs2171838 (posterior probability = 0.72), is located closer to *IRF2*, ~300 Kb of the *IRF2* TSS. This association did not reach the association threshold for use as a causal instrument (posterior probability = 0.72). Examining cis-associations between rs34985265 and all genes within 1 Mb of *IRF2*, *IRF2* is the only gene to show an association with this SNP.

For *RNF13* the primary instrument, rs9853321 (posterior probability = 0.81), was located in a peak 400 Kb upstream of the *RNF13* transcription start site. The strongest independent cis-eQTL, rs62282739, is located nearly 1 Mb downstream of *RNF13* and was too weak to be taken froward for causal analysis (posterior probability = 0.70). Cis-associations for rs9853321 in this region, include an association with the gene *TM4SF1* (posterior probability = 0.93) at a higher level than the association with *RNF13*. There is some indication of a causal relationship between *RNF13* and *TM4S1* (posterior probability = 0.73), however *TM4S1* is not a target of *RNF13* at either a 10% or 15% global FDR threshold, suggesting that *TM4S1* is independently associated with rs9853321.

The third subcutaneous fat sub-network was predicted using the SNP rs35571244 as a cis-eQTL for *PBX2* (posterior probability = 0.93). This SNP is located ~800 Kb downstream of the *PBX2* transcription start site and is the strongest cis-eQTL for a peak of SNPs in this region. An alternate cis-eQTL, rs3128947 (posterior probability = 0.73), is located 500 Kb upstream of the *PBX2* transcription start site. Again, this cis-eQTL was too weak to be taken forward for causal analysis. There are 31 cis-associations between rs35571244 and genes within a 1 Mb window of *PBX2* at a 15% FDR threshold (posterior probability > 0.8), of which *PBX2* is the 7th strongest association. Of these cis associations, 10 are causal targets of *PBX2* when using rs35571244 as a genetic instrument at a 15% FDR threshold and 4 are targets at a 10% FDR threshold. This

suggests that these genes are not independently linked to rs35571244, which raises the possibility that these targets would be predicted from other cis-genes and not just *PBX2* specifically.

The primary instrument used to reconstruct the *CD163* sub-network in visceral abdominal fat, rs7954905 (posterior probability = 0.86), is located less than 100 Kb downstream of *CD163*. The strongest independent cis-eQTL, rs2377237 (posterior probability = 0.72), is located ~500 Kb upstream of the *CD163* transcription start site, however this SNP was below the threshold for use as a causal instrument. There were 6 cis-associations at a 15% FDR threshold (posterior probability > 0.78). One of these cis-genes is a target of *CD163* (posterior probability = 0.86) at a 15% FDR threshold, but no genes are targets at a 10% FDR threshold. This target gene is *CD163L1*, which is a paralog of *CD163* located downstream of of *CD163*. The peak represented by rs7954905 is located in the *CD163L1* gene body. *CD163L1* arose as a gene duplication of *CD163* and colocalises with *CD163*[13].

The primary instrument used to reconstruct the *LUC7L3* sub-network, rs6504682 (posterior probability = 0.8), is located within the *LUC7L3* gene body. An independent cis-eQTL, rs2412130 (posterior probability = 0.7) is located in a peak ~1000 Kb upstream of *LUC7L3*. Again, this alternate cis-eQTL did not meet the threshold for use as an instrument. There is only one other cis-gene associated with rs6504682, *ANKRD40* (Findr score = 0.81), however this gene is not a target of *LUC7L3* in either the 15% or 10% FDR causal networks in visceral adipose.

S3 Supplementary figures

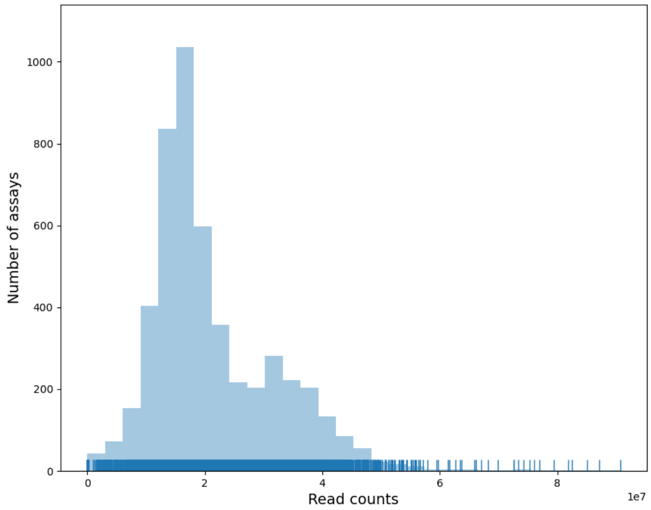

Figure S1: Distribution of RNA-seq read counts across all STARNET tissues.

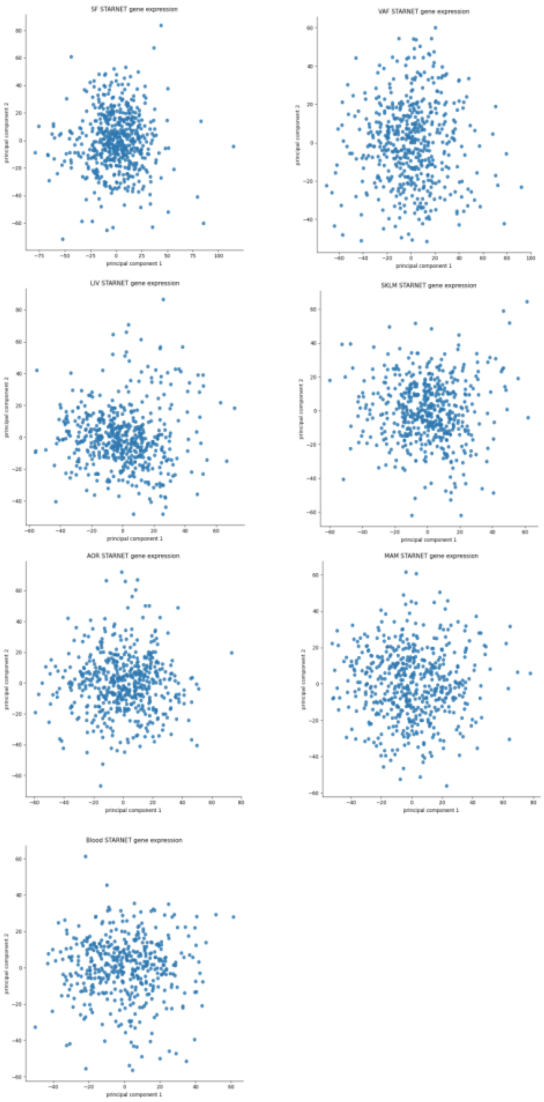

Figure S2: Principal component analysis of gene expression samples across all STARNET tissues.

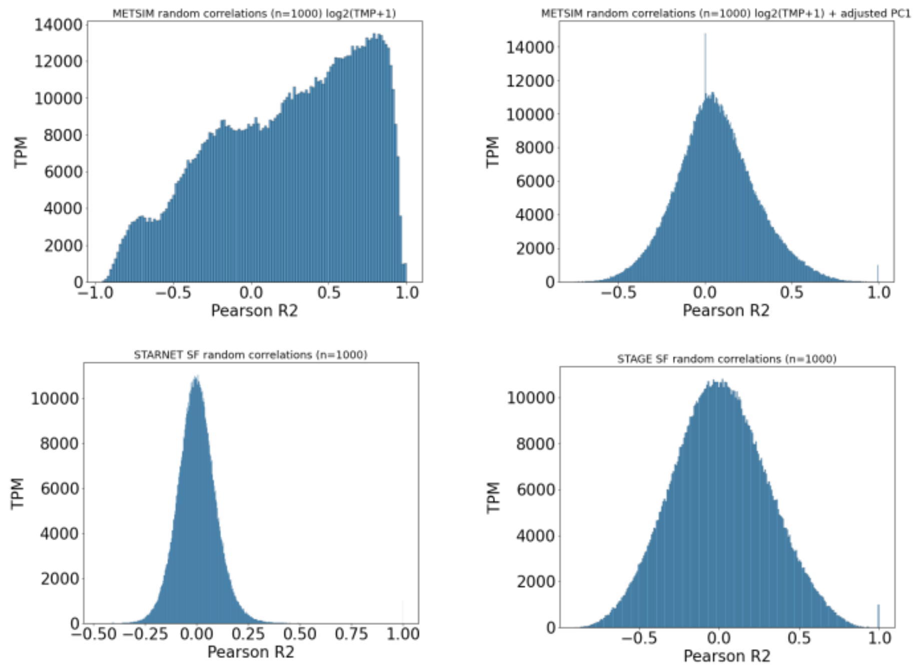

Figure S3: Correlations between randomly sampled genes from discovery and replication datasets both pre and post correction.

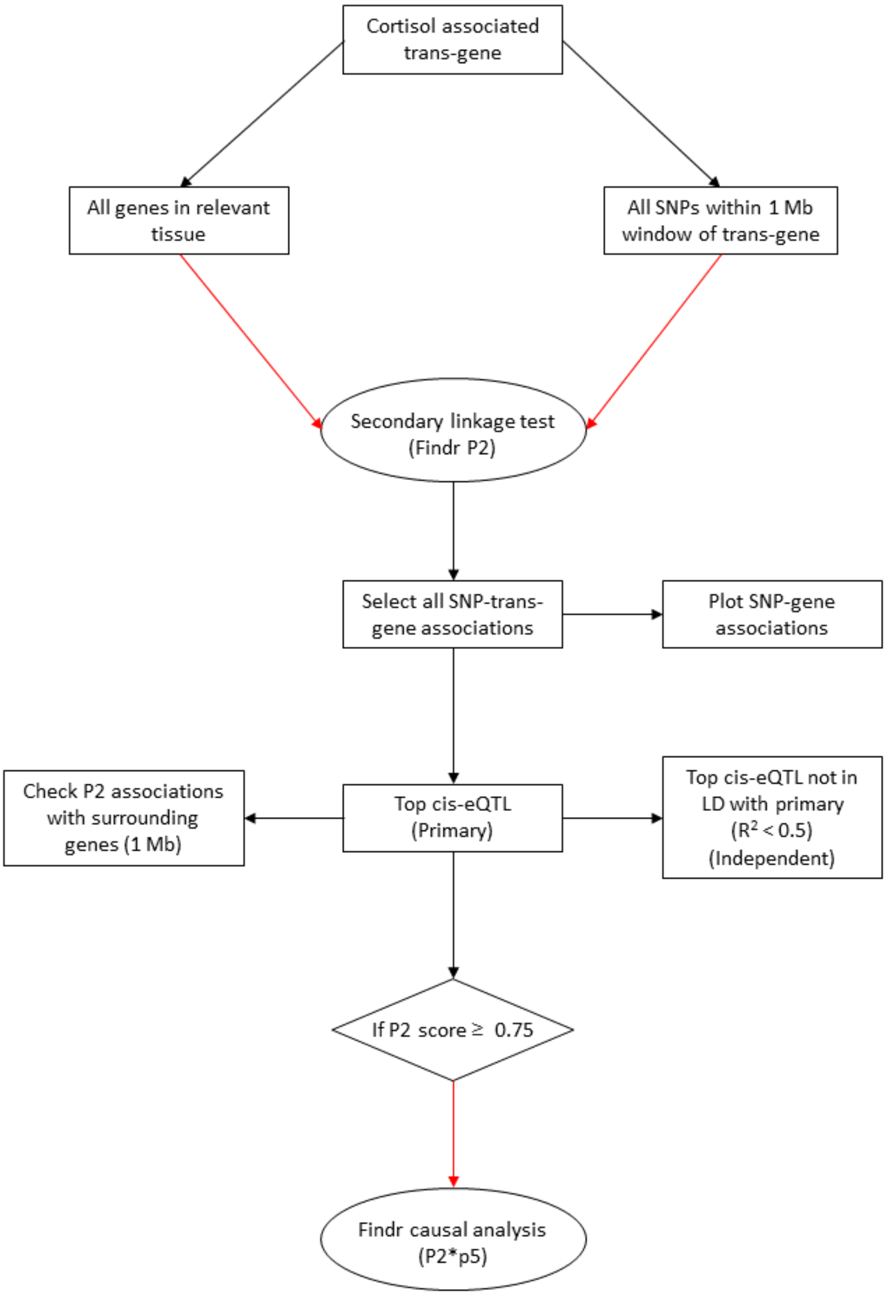

Figure S4: Instrument selection for causal analysis with Findr. Flowchart depicts identification of cis-eQTLs for use as genetic instruments for causal analysis with Findr.

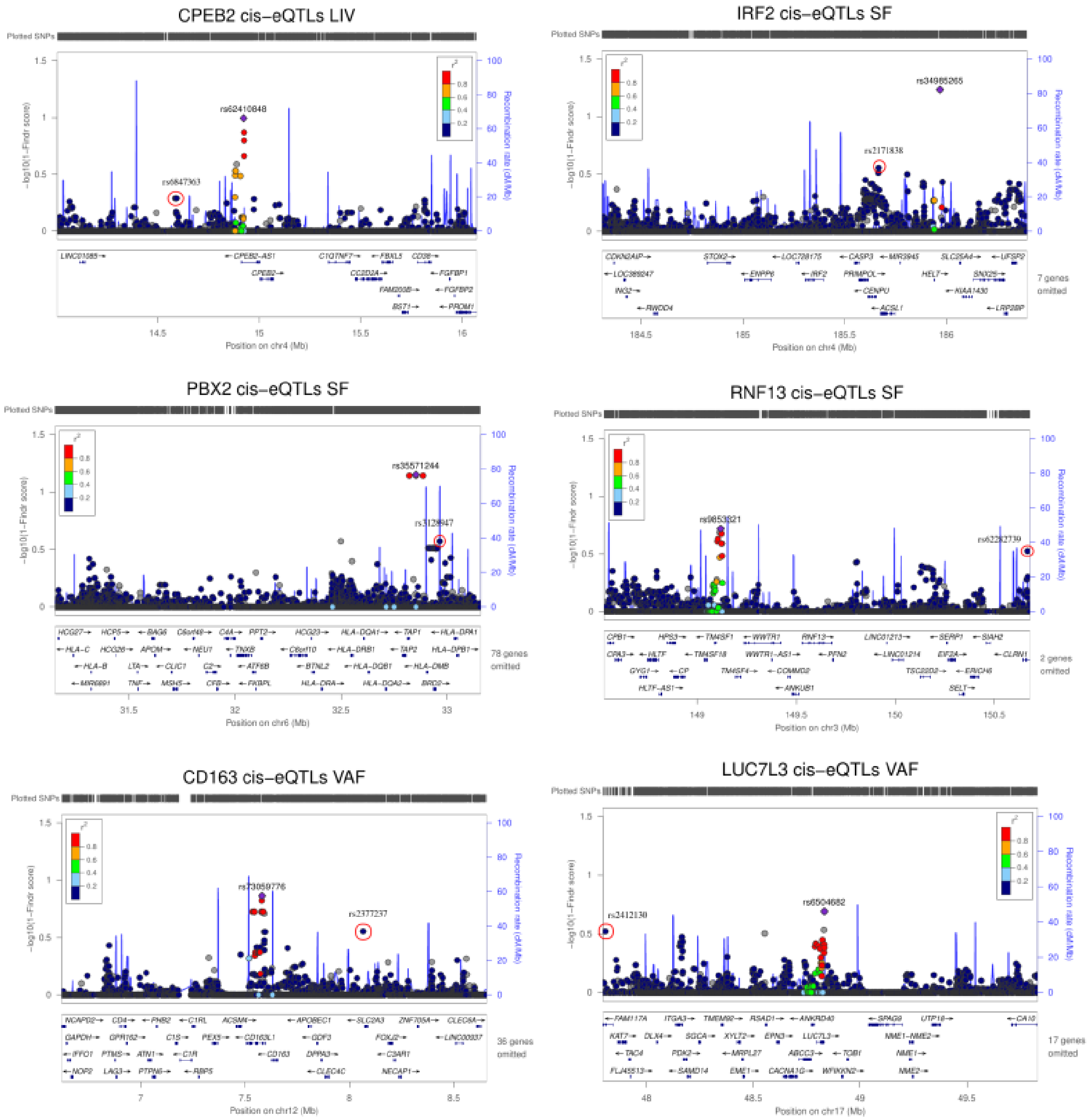

Figure S5: Cis-eQTL discovery for network regulators. SNP-gene associations within a 1 Mb window of the associated gene calculated using the Findr secondary linkage test (P2) and presented as 1-findr score (-$\log_{10}$) with LocusZoom. Lead cis-eQTL is primary instrument used for causal analysis. Red circle indicates independent ($R^2 < 0.5$) alternate instrument.

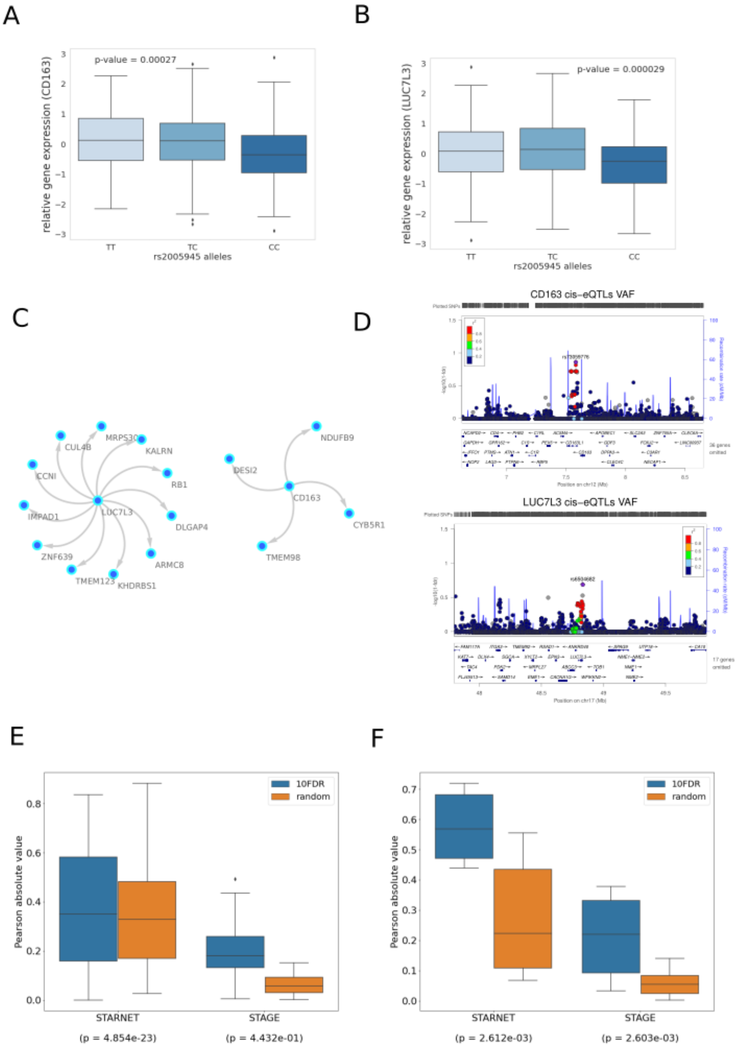

Figure S6: 10% FDR gene network in STARNET visceral abdominal fat driven by *LUC7L3* and *CD163*. (**A**) Gene expression boxplot in STARNET visceral abdominal fat showing trans-association with cortisol-linked SNP rs20005945 and *CD163* (**B**) and *LUC7L3* (p-value obtained from Kruskal Wallis test statistic). **(C)** Causal gene network reconstructed from pairwise interactions from GR-regulated trans-genes against all other STARNET visceral abdominal fat genes. Edges represent Bayesian posterior probabilities of pairwise interaction between genes (nodes) exceeding 10% global FDR. Arrow indicates direction of regulation and interactions were only retained where parent node had at least 4 targets. **(D)** LocusZoom plot showing cis-eQTLs for *CD163* and *LUC7L3*, with lead SNP used as instrumental variable indicated in purple. Significance of association is indicated on the y axis as -log10(1-fdr), where fdr represents the local false discovery rate as estimated by Findr. **(E)** Correlations between network targets in discovery vs replication datasets for *LUC7L3* and **(F)** *CD163*. Kruskal Wallis test calculated for distribution of correlations between network targets compared to correlations within random gene set of same size.

S4 Supplementary tables

Table S1: Datasets used for identifying genes regulated by glucocorticoids.

Table S2: All genes associated with variation for plasma cortisol across all STARNET tissues (FDR = 15%). Only unique associations are included with the top SNP-gene pair. Number of associations refers to the total number of cortisol associated SNPs associated with a given gene.

Table S3: Tissue specific local precision FDR (Findr P2 scores) used to establish FDR thresholds for trans-gene sets.

Table S4: Cortisol associated trans-genes from STARNET-liver (FDR = 15%) with evidence of GR regulation. Transcription factor db includes ENCODE, TRANSFAC and CHEA transcription factor datasets.

Table S5: Cortisol associated trans-genes from STARNET-visceral adipose fat (FDR = 15%) with evidence of GR regulation. Transcription factor db includes ENCODE, TRANSFAC and CHEA transcription factor datasets. ChIP-seq and Microarray fields are from Yu et al experiments in adipocytes[14]. * Indicates genes that have been identified as GR targets from both global TF binding and perturbation experiments. Direction of effect is estimated from the Pearson correlation coefficient of the gene expression level and cortisol associated genotype.

Table S6: Cortisol associated trans-genes from STARNET-subcutaneous fat (FDR = 15%) with evidence of GR regulation. Transcription factor db includes ENCODE, TRANSFAC and CHEA transcription factor datasets. ChIP-seq and Microarray fields are from Yu et al experiments in adipocytes[14]. Murine dex is from dexamethasone treated adrenalectomised mice[15]. Indicates genes that have been identified as GR targets from both global TF binding and perturbation experiments. Direction of effect is estimated from the Pearson correlation coefficient of the gene expression level and cortisol associated genotype.

Table S7: GR regulated cortisol linked trans-genes (FDR = 15%) with a valid cis-eQTL for causal analysis (posterior probability > 0.75).

Table S8: Functional enrichment of causal network targets using DAVID. Filtered to enrichment score > 1.

Table S9: All pairwise interactions from Findr (P2*P5) in liver at a 10% FDR threshold.

Table S10: All pairwise interactions from Findr (P2*P5) in subcutaneous fat at a 10% FDR threshold.

Table S11: All pairwise interactions from Findr (P2*P5) in visceral abdominal fat at a 10% FDR threshold.

Table S12: Transcription factor enrichment within gene network targets. Fisher's exact test for *IRF2* targets from TRANSFAC predicted targets and GR targets from ENCODE. STARNET subcutaneous fat genes used as background for enrichment.

Table S13: Summary of tissue specific gene expression data from STARNET.

References

[1] Franzén, Oscar et al. "Cardiometabolic risk loci share downstream cis- and trans-gene regulation across tissues and diseases". en. *Science* 353.6301 (2016), pp. 827–830.

[2] Wang, Lingfei and Michoel, Tom. "Efficient and accurate causal inference with hidden confounders from genome-transcriptome variation data". *PLOS Computational Biology* 13.8 (2017), e1005703.

[3] Tong, Pin, Monahan, Jack, and Prendergast, James G. D. "Shared regulatory sites are abundant in the human genome and shed light on genome evolution and disease pleiotropy". en. *PLOS Genetics* 13.3 (2017), e1006673.

[4] Ludl, Adriaan-Alexander and Michoel, Tom. "Comparison between instrumental variable and mediation-based methods for reconstructing causal gene networks in yeast". en. *Molecular Omics* 17.2 (2021), pp. 241–251.

[5] Chen, Lin S., Emmert-Streib, Frank, and Storey, John D. "Harnessing naturally randomized transcription to infer regulatory relationships among genes". *Genome Biology* 8.10 (2007), R219.

[6] Huang, Da Wei, Sherman, Brad T., and Lempicki, Richard A. "Systematic and integrative analysis of large gene lists using DAVID bioinformatics resources". en. *Nature Protocols* 4.1 (2009), pp. 44–57.

[7] Kamijo, T., Aoyama, T., Komiyama, A., and Hashimoto, T. "Structural Analysis of cDNAs for Subunits of Human Mitochondrial Fatty Acid β-Oxidation Trifunctional Protein". en. *Biochemical and Biophysical Research Communications* 199.2 (1994), pp. 818–825.

[8] IJlst, Lodewijk, Wanders, Ronald J. A., Ushikubo, Sciichi, Kamijo, Takehiko, and Hashimoto, Takashi. "Molecular basis of long-chain 3-hydroxyacyl-CoA dehydrogenase deficiency: identification of the major disease-causing mutation in the α-subunit of the mitochondrial trifunctional protein". en. *Biochimica et Biophysica Acta (BBA) - Lipids and Lipid Metabolism* 1215.3 (1994), pp. 347–350.

[9] Sims, H. F. et al. "The molecular basis of pediatric long chain 3-hydroxyacyl-CoA dehydrogenase deficiency associated with maternal acute fatty liver of pregnancy". en. *Proceedings of the National Academy of Sciences* 92.3 (1995), pp. 841–845.

[10] Shang Ming-Mei et al. "Lim Domain Binding 2". *Arteriosclerosis, Thrombosis, and Vascular Biology* 34.9 (2014), pp. 2068–2077.

[11] Bujalska, I. J. et al. "Expression profiling of 11beta-hydroxysteroid dehydrogenase type-1 and glucocorticoid-target genes in subcutaneous and omental human preadipocytes". eng. *Journal of Molecular Endocrinology* 37.2 (2006), pp. 327–340.

[12] Kang, Bit Na, Jude, Joseph A., Panettieri, Reynold A., Walseth, Timothy F., and Kannan, Mathur S. "Glucocorticoid regulation of CD38 expression in human airway smooth muscle cells: role of dual specificity phosphatase 1". *American Journal of Physiology-Lung Cellular and Molecular Physiology* 295.1 (2008), pp. L186–L193.

[13] Moeller, Jesper B. et al. "CD163-L1 Is an Endocytic Macrophage Protein Strongly Regulated by Mediators in the Inflammatory Response". en. *The Journal of Immunology* 188.5 (2012), pp. 2399–2409.

[14] Yu, Chi-Yi et al. "Genome-Wide Analysis of Glucocorticoid Receptor Binding Regions in Adipocytes Reveal Gene Network Involved in Triglyceride Homeostasis". en. *PLOS ONE* 5.12 (2010), e15188.

[15] Bell, Rachel M. B. et al. “Carbonyl reductase 1 amplifies glucocorticoid action in adipose tissue and impairs glucose tolerance in lean mice”. en. *Molecular Metabolism* 48 (2021), p. 101225.
